# The pathological Trento variant of alpha‐1‐antitrypsin (E75V) shows nonclassical behaviour during polymerization

**DOI:** 10.1111/febs.14111

**Published:** 2017-06-08

**Authors:** Elena Miranda, Ilaria Ferrarotti, Romina Berardelli, Mattia Laffranchi, Marta Cerea, Fabrizio Gangemi, Imran Haq, Stefania Ottaviani, David A. Lomas, James A. Irving, Annamaria Fra

**Affiliations:** ^1^ Department of Biology and Biotechnologies ‘Charles Darwin’ Sapienza University of Rome Italy; ^2^ Department of Internal Medicine and Therapeutics Pneumology Unit University of Pavia Italy; ^3^ Department of Molecular and Translational Medicine University of Brescia Italy; ^4^ UCL Respiratory and the Institute of Structural and Molecular Biology University College London UK; ^5^ Center for Diagnosis of Inherited Alpha 1‐Antitrypsin Deficiency Pneumology Unit Fondazione IRCCS Policlinico San Matteo Pavia Italy

**Keywords:** alpha‐1‐antitrypsin deficiency, emphysema, misfolding diseases, serpin polymers, serpins

## Abstract

Severe alpha‐1‐antitrypsin deficiency (AATD) is most frequently associated with the alpha‐1‐antitrypsin (AAT) Z variant (E342K). ZZ homozygotes exhibit accumulation of AAT as polymers in the endoplasmic reticulum of hepatocytes. This protein deposition can lead to liver disease, with the resulting low circulating levels of AAT predisposing to early‐onset emphysema due to dysregulation of elastinolytic activity in the lungs. An increasing number of rare AAT alleles have been identified in patients with severe AATD, typically in combination with the Z allele. Here we report a new mutation (E75V) in a patient with severe plasma deficiency, which we designate Trento. In contrast to the Z mutant, Trento AAT was secreted efficiently when expressed in cellular models but showed compromised conformational stability. Polyacrylamide gel electrophoresis (PAGE) and ELISA‐based analyses of the secreted protein revealed the presence of oligomeric species with electrophoretic and immunorecognition profiles different from those of Z and S (E264V) AAT polymers, including reduced recognition by conformational monoclonal antibodies 2C1 and 4B12. This altered recognition was not due to direct effects on the epitope of the 2C1 monoclonal antibody which we localized between helices E and F. Structural analyses indicate the likely basis for polymer formation is the loss of a highly conserved stabilizing interaction between helix C and the posthelix I loop. These results highlight this region as important for maintaining native state stability and, when compromised, results in the formation of pathological polymers that are different from those produced by Z and S AAT.

**Abbreviations**
AAT: alpha‐1‐antitrypsin
AATD: alpha‐1‐antitrypsin deficiency
ER: endoplasmic reticulum
IEF: isoelectric focusing
PAGE: polyacrylamide gel electrophoresis
RMSF: root‐mean‐square fluctuation

## Introduction

Mutations in the *SERPINA1* gene can cause activity loss and deficiency in the circulating serine protease inhibitor alpha‐1‐antitrypsin (AAT). AAT is primarily secreted by hepatocytes and plays a key role in protecting tissues from proteolytic damage by neutrophil elastase. Alpha‐1‐antitrypsin deficiency (AATD) is most commonly associated with the Z (E342K) variant, which accumulates as ordered aggregates (or ‘polymers’) in the endoplasmic reticulum (ER) of hepatocytes, predisposing to liver disease 1. The consequent low‐circulating levels of AAT result in damage to lung parenchyma and early‐onset emphysema due to uncontrolled activity of neutrophil elastase (2 and reviewed in 3). In addition to liver tissue, polymers are found in the circulation 4, 5 and in lung bronchoalveolar lavage fluid 6, 7, where they are thought to exert proinflammatory effects and thereby worsen pulmonary damage in AATD 8.

In addition to the common S (E264V) and Z variants, several rare alleles have been shown to cause deficiency, normally in association with the Z allele 9, 10, 11. AAT null variants are not detectable in plasma and result from nonsense mutations, splicing mutations or large deletions in the *SERPINA1* gene 12. In contrast, missense mutations lead to synthesis of conformationally unstable AAT variants with a variable tendency to form intracellular polymers and a correlated degree of secretory deficiency. Rare variants exhibiting a severe polymerogenic phenotype include M_malton_ (F52del) 13, S_iiyama_ (S53F) 14 and King's (H334D) 10. Milder polymerogenic properties have been demonstrated for other variants, such as M_wurzburg_, Y_orzinuovi_, P_brescia_ 9, 11 and Baghdad 15. While the frequency of individual rare variants is very low, they collectively account for up to 20% of pathological alleles in Southern European countries 12, 16.

Despite the role of AAT polymer accumulation in the molecular pathology of AATD, the structural mechanism that underpins its formation and the details of the molecular species present in the liver remain a matter of debate. Of several models that have been proposed, the classical ‘loop‐sheet’ model predicates the insertion of the reactive centre loop (RCL) of one molecule into β‐sheet A of another 1. An alternative possibility, based on the crystal structure of a recombinant trimer, involves a domain swap of three C‐terminal β‐strands 17. A third alternative, the β‐hairpin model, has been proposed, in which helix I is unravelled and both strand 5A and the RCL mediate intermolecular contacts 18. However, several lines of evidence have cast doubt on the relevance of this form to pathological polymers in AATD, including observations made using monoclonal antibodies (mAbs). The seminal study that proposed the β‐hairpin model made use of polymers induced by a chemical denaturant 18, and the 2C1 mAb that recognizes polymers present in the liver of ZZ AATD individuals does not recognize those formed under such conditions 17, 19. In addition, the 4B12 mAb recognizes monomer and polymer equally despite an epitope that includes helix I, which is proposed to be displaced in the β‐hairpin model 20.

Pathological mutations in AAT and related serpins, such as PAI‐1 and antithrombin, have a distribution that broadly correlates with patches of conserved residues 21, and collectively demonstrate the importance of specific structural interactions for mechanism and stability 22. The ‘breach’ 1, ‘shutter’ 14, ‘gate’ 22 and ‘latch’ 11 regions, all of which correspond with pathological and subpathological mutations, are directly associated with RCL and central β‐sheet A dynamics during protease inhibition and polymerization.

However, residues responsible for serpin stability are widely distributed throughout the structure 23 and natural variants can help to pinpoint other functionally important regions. Here we report the identification of Trento, a novel AAT variant found in heterozygous association with the Z allele in a patient with severe plasma deficiency. Trento AAT was found to possess an E75V mutation, located in a structural region that has not previously been associated with AATD. It exhibited an unexpected molecular behaviour when characterized in cellular models of disease. Trento produced polymers with an altered immunoreactive and electrophoretic profile, distinct from polymers produced by Z and S AAT, and more consistent with a denaturant‐induced polymer form. Immunological analysis, molecular modelling and patterns of conservation reveal that this is due to the loss of a crucial, highly conserved interaction between helix C and helix I. These data indicate that this region is important for stability against polymerization of AAT.

## Results

### Identification of the AAT Trento variant

The new AAT allele was identified in a 57‐year‐old male current smoker (35 pack‐years) suffering from emphysema. His spirometry values showed obstructive defects with prebronchodilator forced expiratory volume in 1 s (FEV_1_) of 3.51 L (70% of predicted) and forced vital capacity (FVC) of 4.39 L (159% of predicted). His diffusion lung carbon monoxide (DLCO) was reduced (52% of predicted). The AAT plasma levels were determined three times in a period spanning 5 months with a mean value of 22.1 mg·dL^−1^ (± SD: 7.8), consistent with a severe deficiency. C‐reactive protein (CRP) was always within the normal range (mean value 0.04 mg·dL^−1^ ± SD: 0.01). Genetic analysis of the *SERPINA1* gene revealed the presence of the Z mutation in heterozygous association with a novel missense mutation in exon 2 (c296A>T) on an M1Ala background, representing a nonsynonymous glutamic acid‐to‐valine substitution at position 75 (E75V in conventional AAT numbering, equivalent to p.Glu99Val using HGVS guidelines).

**Figure 1:**
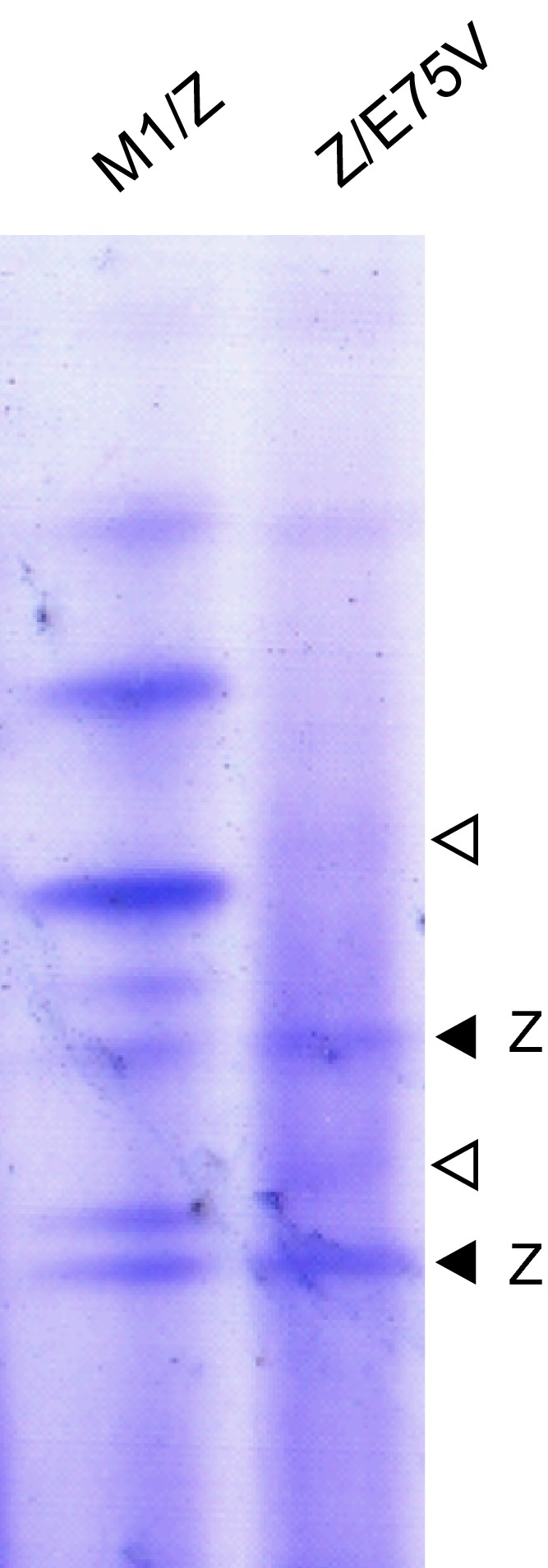
Phenotyping of the AAT Trento variant. An IEF analysis (over the pH range 4.2–4.9) was performed on the proband's plasma compared with that of a known M1Z heterozygote. The main bands corresponding with Z AAT are marked by black arrowheads, while bands corresponding with M AAT are absent from the sample. White arrowheads indicate two weak bands that likely correspond to the Trento AAT mutant.

Neither mutation was present in a brother and a sister, while the parents could not be investigated. AAT phenotyping by isoelectric focusing (IEF) was consistent with a Z phenotype, whereas the E75V variant, which would be expected in the pI range of the S (E264V) variant, was barely detectable (Fig. 1). An absence of bands characteristic of the M variant suggested the presence of compound heterozygosity with two nonwild‐type alleles. After diagnosis, the patient began receiving replacement therapy by weekly intravenous infusion of exogenous AAT. The E75V variant was designated ‘Trento’, according to the birthplace of the proband.

### AAT Trento is mildly deficient in cell culture models of AATD

**Figure 2:**
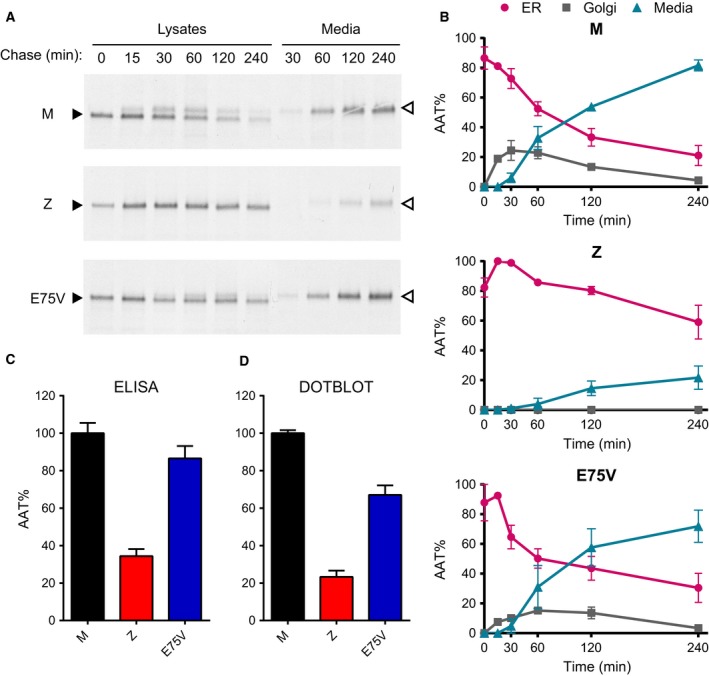
Analysis of Trento AAT secretion in Hepa 1.6 cells. (A) Pulse‐chase experiments on Hepa 1.6 cells expressing M, Z or Trento AAT. Transfected Hepa 1.6 cells were pulsed with ^35^S‐Met/Cys for 10 min and chased for the indicated times. AAT was immunoprecipitated from cell lysates and media and analysed by 7.5% SDS/PAGE and autoradiography. Black arrowheads: immature high‐mannose AAT; white arrowheads: complex *N*‐glycosylated AAT. (B) Densitometric analysis of the autoradiograms of pulse‐chase experiments (*n* = 2). The relative amounts of AAT at the different chase times with an immature ER glycosylation pattern (pink), a Golgi‐associated mature glycan pattern (grey) and present in the cell medium (light blue) are expressed as percentage (mean ± SEM;* n* = 2) of the total amount of intracellular AAT at the beginning of chase, which was set at 100%. (C) Cells expressing M (black), Z (red) or Trento (blue) AAT were cultured for 24 h in the absence of serum and the AAT levels in the media were analysed by ELISA (*n* = 2) or (D) a semiquantitative dot blot followed by densitometric analysis (*n* = 4). For each experiment, the values are expressed as a percentage of M AAT secretion and the graphs represent the means and SEM of replicate experiments.

Wild‐type AAT is synthesized as an immature high‐mannose glycoprotein in the ER, and rapidly matures to the complex N‐glycosylated form in the Golgi with efficient secretion into the culture medium. In contrast, appreciable levels of the Z variant accumulate in the ER as the high‐mannose form, with a concomitantly decreased secretion. The low level of circulating AAT in the proband – similar to that observed in a ZZ homozygote – suggested that the AAT Trento variant may represent a novel Z‐like severe deficiency allele. To investigate its intracellular handling and secretion, we expressed Trento AAT in Hepa1.6 cells and performed pulse‐chase experiments (Fig. 2A). The patterns of intracellular maturation and secretion for wild‐type M and mutant Z AAT variants, measured for comparison, were consistent with those reported previously 24. Quantification of the three variants using densitometry (*n* = 2) revealed kinetics of Trento intracellular N‐glycan maturation and secretion efficiency only slightly lower than those of wild‐type M AAT (Fig. 2B). The percentage of secretion after 240 min of chase, relative to the initial intracellular AAT level, was 81.5 ± 3.7%, 21.7 ± 7.8% and 71.8 ± 10.9% (± SEM) for M, Z and Trento AAT respectively.

The mild secretory deficiency of the AAT Trento mutant observed in the pulse‐chase experiments was confirmed by quantification of AAT in the cell culture media following transfection of Hepa1.6 cells, by both ELISA (*n* = 2) and semiquantitative dot blot analysis (*n* = 4; Fig. 2C,D). This behaviour was independent of the cellular host used for expression: similar results were obtained when the proteins were expressed in COS‐7 (as observed below in Fig. 4) or HEK293T (not shown) cells.

### Confirmation of low Trento AAT levels in patient plasma

**Figure 3:**
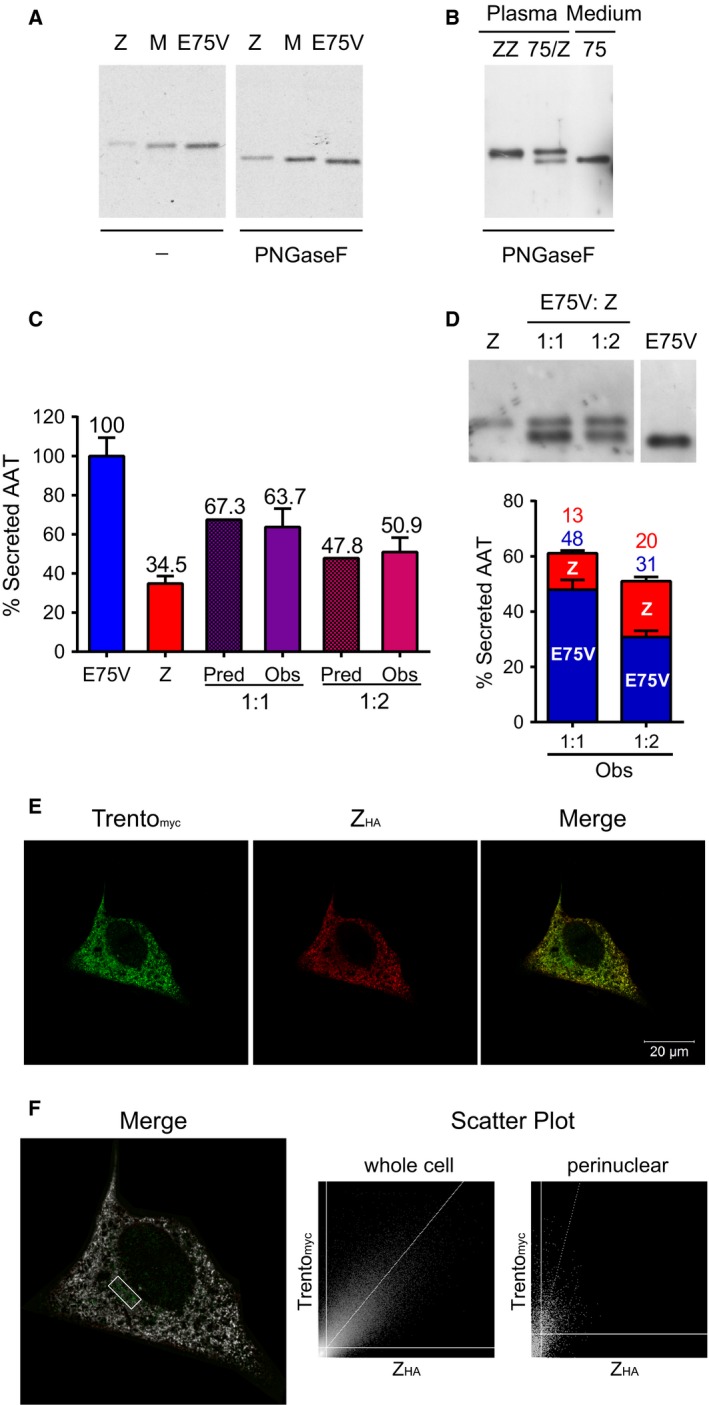
The effect of coexpression with the Z variant on Trento AAT secretion. (A) Different electrophoretic mobilities in 10% SDS/PAGE of pulse‐labelled M, Z and Trento AAT (cell lysates, chase time 0), with or without digestion with PNGase F. (B) Z/Trento plasma was compared by 10% SDS/PAGE and anti‐AAT immunoblot with that of a control ZZ patient and with Trento AAT in cell media, after treatment with PNGase F. (C) Hepa 1.6 cells were transfected with plasmids encoding Trento (blue) and Z (red) AAT alone or mixed at 1 : 1 and 1 : 2 (Trento : Z) ratios (purple/pink), keeping the total amount of transfected DNA constant. Levels of AAT in the cell media were determined by ELISA (*n* = 2) and expressed as percentage of secreted AAT normalized to Trento (mean ± SEM). Observed (Obs) values are compared to the values predicted (Pred) from secreted amounts of the variants expressed alone. One‐way ANOVA *P* < 0.0001. (D) Upper panel: Proteins in the cell media quantified in panel C were deglycosylated with PNGase F and analysed by 11% SDS/PAGE and immunoblot to separate Z and Trento AAT. Lower panel: The ratio of Z (red) and Trento (blue) were determined by densitometry of immunoblots in *n* = 2 experiments and represented in the graph as fractions of secreted AAT (mean ± SEM). (E) Hepa 1.6 cells were cotransfected with plasmids encoding for the tagged variants Trento_myc_ and Z_HA_ and analysed by confocal microscopy. Cells were labelled with a rabbit anti‐myc polyclonal antibody and a mouse anti‐HA mAb; followed by 488 nm ALEXA®‐conjugated anti‐rabbit and 594 nm ALEXA®‐conjugated anti‐mouse secondary antibodies. Micrographs were acquired by the LSM510META confocal microscope (Zeiss) with a EC Plan‐Neofluar 100x/1.30 oil objective. (F) Colocalization was analysed by the Colocalization Threshold Tool in ImageJ 50 and represented in white colour. The scatter plot shows an overall colocalization of the two variants. The Trento variant is also localized in a perinuclear region likely corresponding to the Golgi apparatus, as highlighted in the magnification box and the relative scatter plot.

The efficient secretion of AAT Trento by transfected cells, unexpected in view of the low‐circulating levels detected in the patient, was in clear disagreement with the generally good correlation we observe between plasma AAT concentrations and secretion in our cellular models 9, 10, 11. In our analysis of the pulse‐chase experiments, we observed a faster migration in SDS/PAGE of AAT Trento compared to Z AAT. This difference was more evident following N‐glycan removal by treatment with the glycosidase PNGase F under denaturing conditions (Fig. 3A). As the expression plasmids were identical in every other respect, the marginal but reproducible difference in migration was therefore a direct consequence of the amino acid substitution.

To evaluate the expression of the novel allele in the patient, and exclude artefacts in the nephelometric determination of clinical samples, we took advantage of the different migration of Z and Trento AAT observed in SDS/PAGE (Fig. 3A). We analysed by SDS/PAGE and immunoblot the Z/Trento AAT plasma compared with that from a control ZZ individual and with the supernatant of cells expressing Trento AAT, after treating the samples with PNGase F (Fig. 3B). The proband's plasma showed two distinct AAT bands of similar intensity that comigrated with the Z and Trento AAT bands in the control lanes, confirming a Z‐like circulating level for Trento AAT.

### Z AAT does not exert a dominant retention effect on Trento AAT

It has been observed that the Z AAT variant can copolymerize with other mild deficiency variants of AAT 25. To investigate whether the low level of Trento AAT observed in the proband's plasma was due to a dominant retention effect exerted by Z AAT, we performed coexpression experiments. Hepa 1.6 cells were transfected with plasmids encoding Trento or Z AAT alone, or with plasmid mixes at either 1 : 1 or 1 : 2 ratios, keeping the total amount of transfected DNA constant. Determination of the resulting AAT levels in the cell media by ELISA (*n* = 2) showed that coexpression did not reduce total secreted AAT relative to our prediction based on the levels of the two variants alone. The average value of Trento AAT was set at 100% and the observed percentages were 100 ± 9.4% and 34.5 ± 3.8% (± SEM) for Trento AAT and Z AAT alone, and 63.7 ± 9.4 and 50.9 ± 7.4 for the 1 : 1 and 1 : 2 coexpression conditions respectively (Fig. 3C).

The cell media supernatants were then analysed by SDS/PAGE and immunoblot after deglycosylation with PNGase F in order to distinguish between secreted Z AAT and Trento AAT (Fig. 3D, upper panel). The proportion of Trento AAT and Z AAT in the secreted material analysed in Fig. 3C was quantified by densitometric analysis of the bands of both variants in two distinct experiments (Fig. 3D, lower panel). The absence of substantially reduced secretion of Trento during coexpression with Z AAT makes this unlikely to be the cause of the low Trento AAT levels observed in the patient's plasma. Again, this was not cell‐line specific: similar results were observed during coexpression of Trento and Z AAT in HEK293T cells (data not shown).

Taken as a whole, the secretion data are consistent with a protein that folds and is secreted at near wild‐type levels, in contrast to the Z variant which is known to exhibit delayed folding 26 and compromised secretion. The intracellular localization of Trento and Z AAT, respectively, modified with ‐myc and ‐HA tags, was also studied by confocal immunofluorescence microscopy (Fig. 3E), and showed an enrichment of Trento AAT in a region most probably corresponding to the Golgi apparatus when compared to Z AAT (Fig. 3F), consistent with its more efficient secretion.

### Trento AAT forms polymers with reduced reactivity to conformation‐selective antibodies

**Figure 4:**
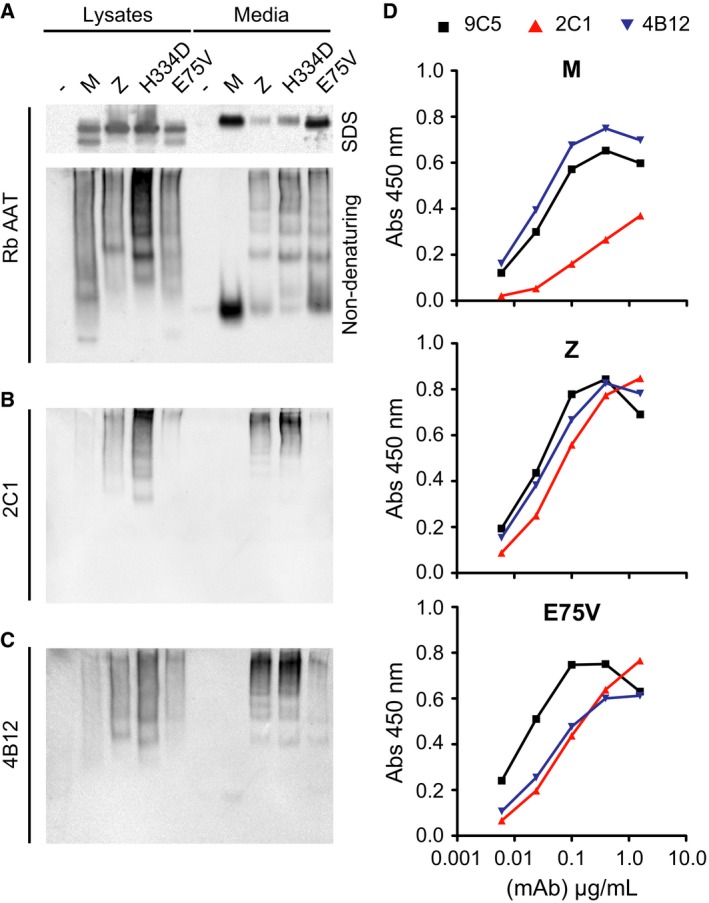
Polymers of Trento AAT are differentially recognized by conformation‐specific mAb. (A) COS‐7 cells were transiently transfected with the AAT variants as indicated, and the soluble cell lysate and culture media collected after 48 h were resolved by SDS or nondenaturing PAGE, followed by western blot with a rabbit polyclonal anti‐AAT (Rb AAT). (B) The same membrane in (A) for the nondenaturing PAGE was immunostained with mAb 2C1 (anti‐AAT polymer). (C) Equivalent samples to (A) and (B) were resolved in a parallel membrane and probed with mAb 4B12. (D) Affinity curves of three mAb [9C5 (black), 2C1 (red) and 4B12 (blue)] by sandwich ELISA, using culture medium supernatants of COS‐7 cells transfected as in (A) with M, Z or Trento AAT as the antigens. The graphs shown are representative of three independent experiments with similar results.

To investigate whether the new AAT variant forms polymeric structures, we expressed the protein in COS‐7 cells, using M, Z and the highly polymerogenic King's AAT (H334D) 10 for comparison. The intracellular lysates and culture media of transfected cells were analysed by SDS‐ and nondenaturing PAGE followed by immunoblot with a rabbit anti‐AAT polyclonal antibody that recognized all conformations of AAT (Fig. 4A). As observed for Hepa1.6 cells, Trento AAT was secreted at higher levels than the severe deficiency Z and King's (H334D) AAT (Fig. 4A, upper panel). Under nondenaturing conditions, both monomeric and higher molecular weight forms were evident for Trento AAT, in common with the Z and H334D variants (Fig. 4A, lower panel). The immunoblot membrane was subsequently reprobed with mAb 2C1, which recognizes polymers isolated from ZZ tissue and heat‐induced polymers to a greater extent than denaturant‐induced polymers 10. This antibody was able to specifically recognize a polymeric ladder in cell lysates and culture supernatants, albeit to a noticeably lesser degree for Trento AAT relative to the other mutants (Fig. 4B). This effect was particularly prominent for polymers present in the cell media supernatant. AAT Trento polymers were also detected less efficiently by the 4B12 mAb (Fig. 4C), whose epitope spans the A and I helices and recognizes Z AAT polymers 20.

To evaluate the differential recognition of the AAT variants using a more quantitative technique, the affinities of mAbs 2C1 and 4B12 for M, Z and Trento AAT relative to 9C5 (a reference mAb with good affinity for all conformers of AAT 10) were also evaluated by sandwich ELISA (Fig. 4D). These experiments confirmed an approximately 10‐fold decrease in recognition of Trento AAT polymers by both mAb 2C1 and 4B12 with respect to M and Z AAT.

### Localization of the 2C1 epitope to the region between helices E and F

**Figure 5:**
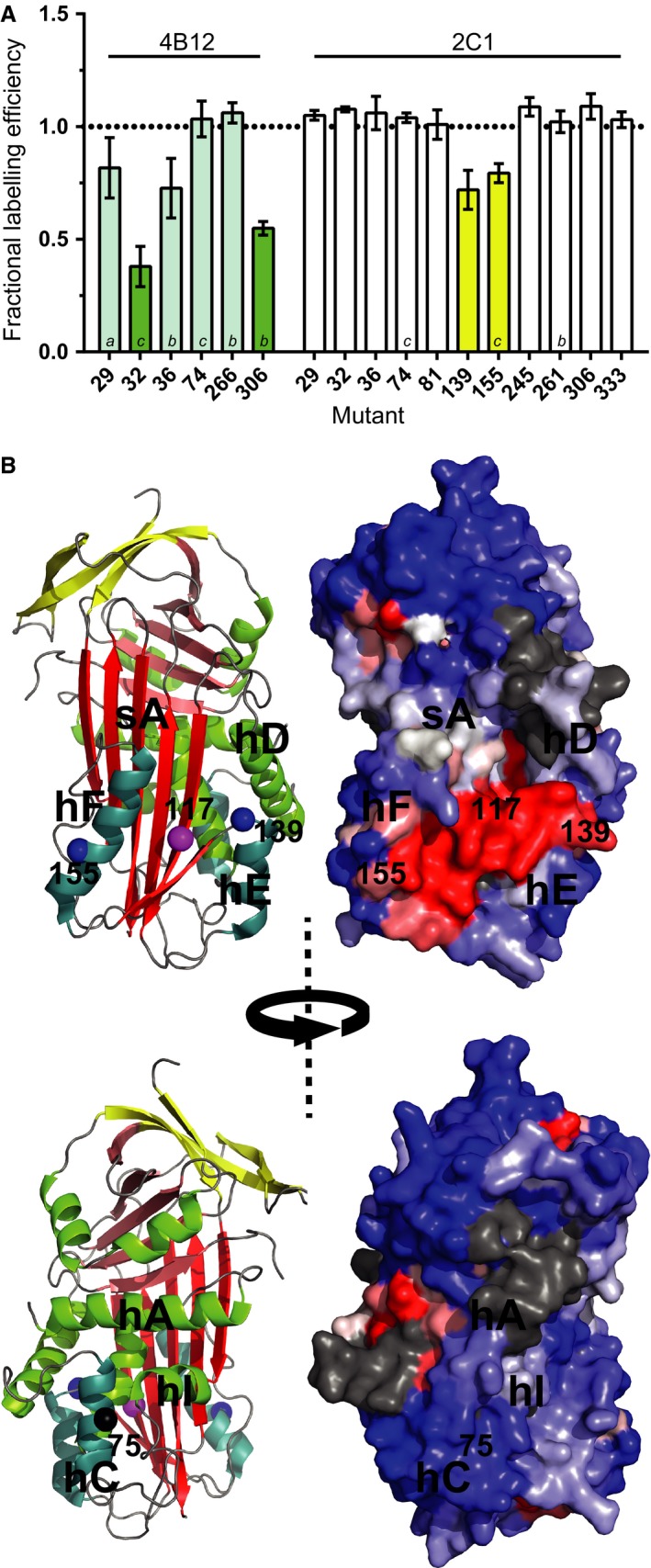
Localization of the mAb 2C1 epitope. (A) Single‐cysteine mutants (0.1 mg·mL ^−1^ final concentration) in the presence and absence of a 0.5 : 1 molar ratio of mAbs 4B12 (cyan/green) or 2C1 (white/blue) were incubated with the CPM dye. The change in fluorescence (excitation 370 nm and emission 490 nm) was monitored for 30 min. The fractional intensity of each curve in the presence of the antibody was calculated with respect to its absence. This property reflects the degree of the exposure of the single cysteine; a lower value in the presence of an antibody is indicative of shielding due to binding. Green bars indicate positions previously identified in the 4B12 epitope; blue bars indicate sites of shielding in the presence of the 2C1 antibody. Values represent mean ± SEM (*n* = 4, except: *a* denotes *n* = 2; *b*,* n* = 3; *c*,* n* = 5). (B) Left panels: Two residues identified by this approach are shown as blue spheres on a cartoon representation of cleaved AAT (PDB accession 1EZX) in addition to the location of the G117F mutation that yields polymers nonreactive with 2C1 10 in magenta and the Trento mutation at 75 is shown in black. Helices C, E and F are in cyan, and β‐sheets A, B and C are in red, pink and yellow respectively. Right panels: a comparison of surface patches between wild‐type M AAT, which produces 2C1‐recognized polymers, and the G117F mutant, which does not. For each surface‐accessible residue on wild‐type recombinant AAT (PDB accession 1QLP), an epitope‐sized surface patch was defined comprised of all positions with a Cα atom within 8 Å of this central site. Each patch was then optimally aligned against identical residues in the crystal structure of G117F (accession 3DRU), the root‐mean‐square deviation between equivalent backbone Cα atoms was calculated, and the value was mapped onto the original central surface‐accessible residue. This method of structural comparison has been described elsewhere 38. Blue denotes the centre of patches with a high degree of similarity between the structures, and red denotes a high degree of structural divergence. Grey indicates patches that overlap glycan attachment sites; as 2C1 binds to glycosylated material, the epitope would not overlap with these positions. The figure was generated using PyMol (Schrodinger).

As the epitope of mAb 2C1 has not yet been determined, it is possible that the E75V mutation could directly alter this epitope and reduce the affinity for Trento AAT polymers. To ascertain this, a protection assay using the thiol‐reactive fluorophore 7‐diethylamino‐3‐(4‐maleimidophenyl)‐4‐methylcoumarin (CPM) was developed to map the binding site of mAb 2C1. This approach was validated using mAb 4B12, whose epitope has been localized using PEGylation and spin‐labelling 20. Monomeric single‐cysteine recombinant mutants were incubated with the 4B12 antibody, the CPM fluorophore was added, and the increase in fluorescence due to thiol conjugation was followed over time. Increased protection of a cysteine against conjugation due to the binding of the antibody resulted in a reduced labelling efficiency and a concomitant decreased overall fluorescence. The resulting curves exhibited good correspondence with the previously determined 4B12 epitope, including a lack of interaction at position 74 on helix C (Fig. 5A, left, shaded bars). This suggests that reduced binding of the 4B12 antibody to Trento AAT is not due to direct effects by the E75V mutation on the epitope, but rather through indirect structural changes.

For experiments with mAb 2C1, the single‐cysteine mutants were subjected to heat‐induced polymerization prior to incubation with the antibody. However, mutations near E75, including 29, 74 and 81, showed no effect, while 2 of 11 mutants surveyed – 139 and 155 – exhibited a reduced degree of fluorophore conjugation in the presence of 2C1 (Fig. 5A, right, white bars). This is consistent with an earlier observation 10 in which a stabilizing G117F mutant was noted to form polymers that were not recognized by 2C1: positions 139 and 155, on helix E and F respectively, flank this site (Fig. 5B, left panels). The G117F mutation leads to a network of structural changes; comparison of surface patches of G117F to the wild‐type recombinant protein reveals the greatest surface difference between these two variants occurs on the face bounded by helices E and F (Fig. 5B, right panels). Further support for this epitope localization comes from a study in which a disulphide bond engineered to restrict movement of helix F resulted in polymers that were also only marginally detectable by 2C1 27. These collective data are consistent with an epitope that lies within the helix E/F interhelical region. This region is notable for undergoing change during the five‐ to six‐stranded transition of β‐sheet A, and is the site of binding of antagonists of conformational change – vitronectin by PAI‐1 28 and an N‐terminal extension by tengpin 29. In regard to Trento AAT, the position of the mAb 2C1 epitope on the opposite side to helix C (Fig. 5B, lower panels) indicates that the decreased reactivity with the antibody is not due to direct effects of the E75V mutation on binding. This makes it likely that 2C1 is reporting a change in the nature of the polymer itself.

### The oligomeric pattern of Trento AAT resembles polymers prepared *in vitro* under denaturing conditions

The pathway of polymer formation has multiple steps and several alternative outcomes. Under certain conditions, different intermediates 30, 31, 32, polymer states 17, 19, 33 and the latent conformation 34, 35 are potentially accessible during polymerization. One representation of the pathway represents the transition between monomer (M), monomeric intermediate (M*), polymer (P) and the monomeric, inactive latent form (L):


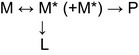


**Figure 6:**
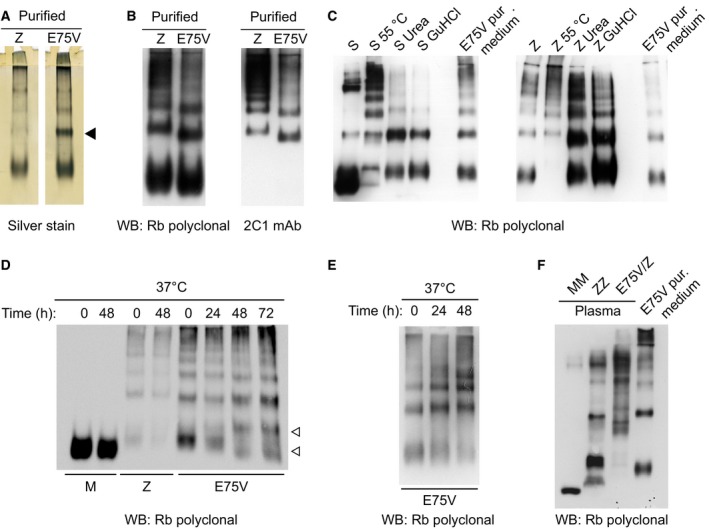
PAGE analysis of the Trento AAT oligomeric pattern. (A–B) Oligomeric profile of Z and Trento AAT, affinity‐purified from cell culture supernatants of HEK293T‐transfected cells, analysed by 7.5% nondenaturing PAGE followed by either silver staining (A) or immunoblot with anti‐total AAT Ab (B, left panel) or 2C1 mAb (B, right panel). (C) Nondenaturing PAGE comparison of Trento purified from culture medium with S or Z AAT purified from plasma, polymerized *in vitro* either by heating at 55 °C, or by incubating with 3 m guanidinium HCl (GuHCl) or 3 m urea. (D) Cell culture media collected after 48 h from COS‐7 transfected cells were additionally incubated for the indicated times at 37 °C and analysed by nondenaturing PAGE. (E) Purified Trento AAT incubated for the indicated times at 37 °C and analysed by native PAGE and immunoblot. (F) Nondenaturing PAGE and immunoblot comparison of Trento AAT purified from culture medium with total plasma of MM, ZZ or E75V/Z.

A difference in the affinity of conformation‐specific mAbs for polymers of AAT Trento is a reflection of structural changes in their respective epitopes. These changes may be associated with altered rates and preferred species along the polymerization pathway, such as at the monomer activation and monomer addition steps. The electrophoretic profile following separation by nondenaturing PAGE can provide a sensitive measure of this; heat‐induced polymers exhibit a distribution enriched for high molecular weight oligomers, whereas treatment with denaturants results in a preponderance of lower molecular weight species 19, 32, 36, 37. Z and Trento AAT were purified from cell culture media of transfected HEK293T cells by affinity chromatography and analysed by nondenaturing PAGE, with visualization by silver staining (Fig. 6A) or immunoblotting (Fig. 6B). Although Z AAT showed an accumulation of higher molecular weight bands that were well recognized by mAb 2C1 (also seen in Fig. 4), AAT Trento favoured a dimeric form that was less reactive to mAb 2C1 than higher order oligomers.

The electrophoretic profile obtained for Trento AAT exhibited the hallmarks of AAT treated with denaturing agents. To confirm this, we compared the migration by nondenaturing PAGE of AAT Trento purified from cell media to that of Z and S AAT purified from plasma samples and polymerized either by heating for 4 h at 55 °C or by exposing for 30 h at room temperature to 3 M urea or 3 M guanidinium hydrochloride (Fig. 6C). Although the profile of Z AAT resembled higher molecular weight heat‐induced polymers for which 2C1 shows high affinity, that of Trento AAT was more similar to the pattern of low molecular weight polymers of Z and S AAT prepared in the presence of denaturants.

### Trento AAT is conformationally unstable at physiological temperatures

To investigate the stability of the mutant proteins under physiological conditions, we collected cell media supernatant from COS‐7 cells transfected with M, Z and Trento AAT for 48 h, and incubated it in the absence of cells at 37 °C for 0, 24, 48 or 72 h. The samples were analysed by nondenaturing PAGE followed by immunoblot (Fig. 6D). In contrast to M and Z AAT, monomeric Trento AAT was found to convert into slower and faster migrating bands in the vicinity of the original monomeric species (Fig. 6D, white arrowheads), indicative of alternative conformations 19, 34, 35. The latter band is likely to be the latent species, a monomeric conformation in which the protein has become inactivated through incorporation of the RCL into β‐sheet A without cleavage 34. Under these conditions, we did not observe an increase in polymeric forms, possibly due to the low concentration of the mutant protein in the culture medium supernatants and stabilizing factors that are known to be present in the medium 38. We therefore repeated the experiment by incubating affinity‐purified Trento AAT at 37 °C for 24 and 48 h in PBS (Fig. 6E). We observed a reduction in the intensity of the monomer band and an increase in the oligomer species. The presence of polymeric species was confirmed by native PAGE analysis of the proband's plasma (Fig. 6F). The latent and polymeric forms reflect two outcomes that can arise from the common process of activation to polymer‐prone intermediate 39. Both these experiments therefore show the native state instability of Trento AAT at physiological temperature.

### The side chain of E75 is a molecular spacer critical for maintaining native contacts

**Figure 7:**
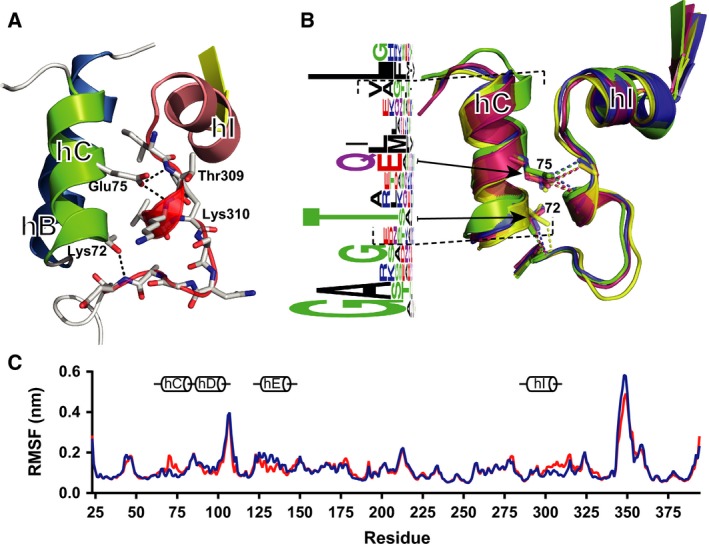
Structural characterization of the E75V mutation. (A) Structural detail of the vicinity of the E75 residue, with helix B in blue, helix C in green, helix I in pink and the posthelix I loop in transparent red, showing the polar interactions mediated by E75 and T72. (B) A comparison of antitrypsin (1QLP, green), neuroserpin (3FGQ, magenta), plasminogen activator inhibitor‐1 (3Q02, yellow) and a *Thermobifida fusca* bacterial serpin (1SNG, blue), showing the evolutionarily and geometrically conserved interaction between Glu75 and the backbone of residues 309–310 (AAT numbering). Patterns of conservation within helix C are represented to the left using Weblogo 51, calculated from the PFAM ‘seed’ alignment of the serpin superfamily (PF00079), after indels were manually adjusted to account for secondary structure. (C) RMSF of backbone atoms averaged over 10 independent 10‐ns all‐atom simulations at 310K of M (blue) and Trento AAT (red), with cylinders highlighting the structural elements (in the vicinity of helices C, D, E and I) whose mobility is affected by the presence of the mutation. The figure was generated using PyMol (Schrodinger).

These collective results indicate that Trento AAT folds efficiently in the ER to a conformation that stays partly monomeric and partly becomes oligomeric, is compatible with secretion, and that the monomeric form is unstable. Position 75 is located on helix C, and in the native state, the glutamic acid side chain forms hydrogen bonds with the backbone atoms of T309 and K310, situated C‐terminal to helix I. In combination with a hydrogen bond formed by T72, these interactions stabilize the posthelix I loop (Fig. 7A). A comparison with the crystal structures of other native serpins reveals that this is a strongly geometrically conserved interaction that yields a precise distance between the two helices (Fig. 7B, right). This is highlighted by an analysis of the evolutionary substitution pattern at this position: where a replacement has occurred, this is almost exclusively with a glutamine residue (Fig. 7B, left), which has an identical side‐chain length. The striking absence of chemically similar residues that are one carbon shorter – aspartic acid and asparagine – supports a role for this position as a molecular ‘spacer’ between helix C and the posthelix I loop, with a critical length and orientation.

This high degree of conservation is strongly supportive of a role for E75 in maintaining a functional inhibitor. Our cell secretion results indicate that, while AAT can adopt an ordered, folded conformation in the absence of this spacer residue, it readily becomes inactivated through polymerization. To explore the structural consequences in the vicinity of the mutation, a molecular dynamics (MD) analysis was performed. For each MD simulation, the root‐mean‐square fluctuation (RMSF) of Cα atoms of all residues was calculated for the wild‐type and for the Trento AAT mutant, in order to analyse how the mobility of different portions of the structure is affected by the mutation. As shown in Fig. 7C, there are two main regions where the mutant has a significantly higher mobility with respect to the wild‐type: the first is on helix C close to the mutation site, involving residues from 70 to 80; the second is on helix I, involving residues from 300 to 320. Additionally, lower RMSF values of the mutant were observed in the region involving residues from 130 to 140. Analysis of hydrogen bonds and salt bridges suggests that the pattern of contacts is altered in the mutant protein (Table 1).

**Table 1:** Summary of key hydrogen bonds during MD simulations. The average number of hydrogen bonds between selected pairs of residues was calculated over the course of the MD simulations of wild‐type M and of Trento AAT variants. The difference between these values as a result of the Trento mutation is shown in the last column

Donor	Acceptor	M	Trento	Difference
Glu/Val 75	Thr 309	1.28	0.00	1.28
Glu/Val 75	Lys 310	1.13	0.00	1.13
Thr 72	Asp 317	0.91	0.57	0.34
Val 311	Leu 329	0.95	0.71	0.24
Asn 116	Ser 140	0.65	0.86	−0.21
His 73	Glu 89	0.07	0.25	−0.19
Ala 70	Asp 317	0.00	0.12	−0.12
Lys 135	Glu 141	0.25	0.36	−0.11
Asp 71	Asp 317	0.00	0.11	−0.11

## Discussion

Managed instability is crucial for the physiological inhibitory mechanism of AAT. During the interaction with elastase, a concerted change occurs in which unfavourable interactions distributed throughout the molecule are resolved, with a rapid transition to an energetically favoured inactive conformation. With involvement of a large proportion of the molecule in the metastable‐to‐stable transition, mutations that promote aberrant inactive conformations show a wide distribution throughout the structure. These mutations, when associated with misfolding and polymerization, can cause circulating deficiency, proteolytic dysregulation, and gain‐of‐function toxicity in affected individuals. Correspondingly, given the complex interplay between serpin stability and function, pathological variants have proven invaluable for identifying regions and interactions of mechanistic and structural importance.

Intracellular polymer formation, whether the cause or a consequence of mutant AAT retention within the ER, is a key pathogenetic mechanism in AATD associated with polymerogenic AAT variants. However, the polymer structure(s) and the polymerization mechanisms *in vivo* have not been elucidated and are still the subject of debate. This is confounded by the observation that different *in vitro* conditions can favour the production of qualitatively different polymers 17, 40. The polymerization pathway therefore passes through ‘decision‐points’ which favour or disfavour particular alternate structural outcomes, although whether some alternate forms ever arise *in vivo* remains uncertain. For example, it has been shown that the experimentally inducible monomeric, inactive latent conformation of AAT is effectively absent from individuals with the pathological Z variant 35. Additionally, the 2C1 antibody that recognizes pathological Z polymers and heat‐induced polymers can immunodeplete all material in a cellular expression system, suggesting an absence of the denaturant‐induced form for Z AAT *in vivo* 10.

The variant we describe here, identified in an individual exhibiting severe AATD, is the first description of a pathological mutation of AAT situated on helix C. An examination of other serpins highlights the coordination by the E75 side chain of a highly conserved geometry between helices C and I through stabilization of the posthelix I loop. Loss of this interaction results in the formation of polymers with a profile distinct from that of Z and heated M AAT. The presence of distinct electrophoretic ‘fingerprints’ for heat‐induced and denaturant‐induced polymers can be observed in publications by several groups 19, 32, 36, 37; however, this is the first description of such behaviour in a natural AAT variant. The polymer size distribution in nondenaturing PAGE, coupled with the relative affinity of the 4B12 and 2C1 antibodies, suggests that polymers formed by Trento AAT likely correspond to denaturant polymers. In contrast, those of the Z variant produced in the same cellular system exhibited heat‐like characteristics; this prior observation 19 has questioned the relevance of experiments performed in the presence of denaturant to pathological and heat‐induced polymers. However, the data presented here indicate that these studies may provide insight into the behaviour of Trento AAT: polymerization of Trento AAT would be expected to proceed via an expanded (rather than compact) molten globule‐like intermediate 31, involve destabilization of helix I 18 and possibly strand 5A 17, and favour dimer addition over monomer addition 32.

The currently proposed models of polymerization all agree that the RCL and β‐sheet A are central to the polymerization mechanism, although they disagree in which capacity. Accordingly, ‘breach’ (such as Z) and ‘shutter domain’ (such as S_iiyama_ and M_malton_) variants have been identified which impact directly on these structural elements 13, 14, 22. The observation that the epitope of 2C1, with its high degree of specificity for polymer, is situated in a region that is indirectly but substantially involved in RCL insertion is consistent with this. In contrast, the location of E75V on helix C is outside of these core functional regions, and its effect is highly likely to be mediated by the loss of the interaction with helix I. The involvement of helix I in conformational change was shown by a study in which subtle helix I mutations were found to alter inhibitory activity 41. Correspondingly, MD simulations show a destabilization of both helices C and I for the Trento AAT mutant. The importance of this region for the process of polymerization is highlighted by a mAb that suppresses polymerization in PAI‐1 with an epitope that includes position 74 on helix C 42, and the 4B12 mAb that reduces AAT polymerization with an epitope that includes position 306 of helix I 20. This is further underscored by the striking conservation of either a glutamic acid or glutamine residue at this site throughout the serpin protein family.

In the proband, which shows very low AAT plasma levels and severe pulmonary manifestations, Trento AAT is expressed in compound heterozygosity with Z AAT. Our observation that the new mutant was only mildly deficient in cellular models prompted us to investigate whether this phenotype was due to a dominant effect of the Z variant. *SERPINA1* alleles are expressed codominantly, and plasma levels of AAT in heterozygous patients are thought to result from the independent contributions of the two variants. However, hetero‐polymerization of the S and Z variants of AAT has been demonstrated by *in vitro* studies 25, and an example of heterotypic interactions has been reported in cells between Z and I (R39C) AAT 24. On this basis, we investigated whether Z AAT coexpression could exacerbate retention of Trento AAT, but this was found not to be the case. Our cellular experiments indicate that instead Trento AAT is secreted at almost wild‐type levels, without the delay seen with Z AAT. This suggests that it is able to fold efficiently to conformations that negotiate export from the ER and transit through the secretory pathway, and the conformational instability is manifested most prominently once in the native state. Our previous studies have shown that polymers of Z AAT form within the ER and are secreted from cultured cells, consistent with an intracellular origin for Z AAT polymers found in human plasma 5. Still, the differences in Trento AAT polymer characteristics shown here leave the question open as to whether extracellular Trento AAT polymers behave in a similar fashion, or whether they form directly after monomer secretion into plasma.

Our results do not explain the marked absence of circulating Trento AAT observed in the patient. It can be speculated that the low levels present are due to the instability of the mutant under physiological conditions: our results suggest that the Trento mutation could facilitate the transition of AAT to latent and/or polymers in plasma, as seen for other serpins like antithrombin 43. There is evidence that the circulating half‐life of polymers is considerably lower than that of the monomeric protein, at < 30 h 4 and 4–6 days 44 respectively. Additionally, latent Trento AAT could be prone to interactions with circulating proteins and/or cells in plasma, which might enhance its clearance. The clearance mechanisms for latent and polymeric AAT have not yet been elucidated, but our results suggest further investigation is warranted, in order to assess turnover of aberrant AAT conformers in the circulation.

## Experimental procedures

### Genetic identification of the E75V mutation and phenotyping

Biochemical and genetic tests to diagnose AATD were performed at the Centre for Diagnosis of Inherited Alpha1‐Antitrypsin Deficiency at Fondazione IRCCS Policlinico San Matteo (Pavia, Italy) with the understanding and written consent of each subject. All methodologies were in accordance with the Declaration of Helsinki and were approved by the local ethics committee. The plasma levels of AAT and CRP were determined by a rate immune nephelometric method assay (Immage Immunochemistry System; Beckman‐Coulter, Milan, Italy). DNA was isolated from whole peripheral blood using a commercial extraction kit (DNA IQ System; Promega, Milan, Italy). Genotyping for detection of the S and Z variants was performed by PCR/RFLP, as previously described 45. The new mutation was identified by sequencing all coding exons (II‐V) of the *SERPINA1* gene (RefSeq: NG_008290) as reported previously 46, using the CEQ 8800 genetic analysis System (Beckman Coulter). The phenotype of the new variant was determined by IEF analysis on plasma samples, comparing it with other plasma of known phenotype in a pH gradient of 4.2–4.9 (Multiphor II Electroforesis System; GE Healthcare BioScience, Milan, Italy), as previously described 46. The clinical data were obtained from direct observation of clinical charts and they are reported in an anonymized form.

### Expression vectors, cell lines and transfections

The pcDNA3.1/Zeo (+) expression vectors encoding for human M1(Val213) and Z AAT have been previously described 47. The Trento mutation (c296A>T) was introduced into the vector encoding M1 AAT by Site‐Directed Mutagenesis Kit, Stratagene, Agilent, Roma, Italy), according to the manufacturer’s instructions, using a 5′‐ctgacactcacgatgtaatcctggagggcct primer and its reverse complement. COS‐7 cell transfection used previously reported pcDNA3.1 plasmids encoding M, Z and H334D AAT 10. The AAT variants were expressed by transient transfection in Hepa 1.6, COS‐7 or HEK293T cell lines (ATCC #1830, #1651 and #11268 respectively) maintained in Dulbecco's modified Eagle's medium (DMEM) with 10% FBS (Sigma‐Aldrich, Milan, Italy). Transfections in COS‐7 cells in six‐well plates were performed by Lipofectamine2000 (Life Technologies, Monza, Italy) according to the manufacturer's protocol, using 4 μg DNA and 10 μL Lipofectamine in serum‐free Optimem (Life Technologies) for 6 h. Hepa1.6 cells and HEK293T cells were transfected by Polyethylenimine ‘Max’ (PEI, PolySciences Inc, Hirschberg, Germany). DNA–‐poly(ethylenimine) complexes were made in serum‐free DMEM (3 μg DNA and 18 μg poly(ethylenimine) in 400 μL for 10 cm^2^ plates) and then added to cells in normal culture medium for 5 h.

### Immunofluorescence and confocal microscopy

For immunofluorescence staining, Hepa 1.6 cells grown on coverslips were cotransfected with plasmids encoding Trento_myc_ and Z_HA_. After 24 h, the cells were fixed with 4% paraformaldehyde and permeabilized with 0.1% Triton X‐100. Staining was performed with a rabbit anti‐myc polyclonal antibody (Sigma‐Aldrich) and a mouse anti‐HA mAb (Sigma‐Aldrich); followed by 488 nm ALEXA®‐conjugated anti‐rabbit and 594 nm ALEXA®‐conjugated anti‐mouse secondary antibodies (Thermo Fisher Scientific, Monza, Italy). Coverslips were mounted with 50% glycerol in PBS and analysed by the LSM510META confocal microscope (Zeiss, Milan, Italy) with a EC Plan‐Neofluar 100×/1.30 oil objective, using the 488 nm or 543 nm lasers in multitrack mode.

### Pulse‐chase experiments

Pulse‐chase experiments were performed as previously described 24. Twenty‐four hours after transfection, Hepa 1.6 cells were pulsed for 10 min with ^35^S Met/Cys (EasyTag™ Express Protein Labelling mix; Perkin Elmer, Milan, Italy) and chased for 0, 15, 30, 60, 120 or 240 min. At each time point, cell culture media were collected and the cells were lysed in a buffer containing 50 mm Tris/HCl (pH 7.4), 150 mm NaCl, 1% NP40, 10 mm *N*‐ethylmaleimide and protease inhibitors (Sigma‐Aldrich). The cell lysates were centrifuged at 3000 ***g***. AAT in the cell lysates and culture media were immunoprecipitated using anti‐AAT polyclonal antibodies (DAKO, Milan, Italy) and PGAgarose (Thermo Fisher Scientific), and then analysed by 7.5% SDS/PAGE followed by autoradiography.

### SDS/PAGE, nondenaturing PAGE and western blot analysis

SDS/PAGE and nondenaturing PAGE of cell lysates and media were performed as previously described 11, 48. Membranes were probed with either a commercial anti‐AAT polyclonal (DAKO), the 2C1 mAb 10 or the 4B12 mAb 38, and then revealed with HRP‐conjugated secondary antibodies (Sigma‐Aldrich) and ECL (EuroClone S.P.A., Milan, Italy).

### PNGase F treatment

Digestion with PNGase F (New England BioLabs, through Euroclone S.P.A.) was performed according to the manufacturer's protocol on undiluted cell media or on plasma samples diluted 1 : 200 with PBS. The samples were denatured at 94 °C for 10 min in denaturing buffer/DTT, then diluted in 50 mm phosphate buffer pH 7.5 containing 1% NP40 and incubated for 1 h at 37 °C.

### Expression and purification of AAT variants from cell media or plasma

For purification of Trento and Z AAT from cell media, HEK293T cells were transfected with poly(ethylenimine) and incubated in serum‐free DMEM for 36 h. The conditioned medium was collected, centrifuged for 5 min at 3000 ***g*** **,** and AAT was purified by affinity chromatography on Alpha‐1 Antitrypsin Select (GE Healthcare). About 36 mL of cell supernatant was loaded onto 500 μL of matrix, washed with 150 mm NaCl/20 mm Tris pH 7.4 and eluted with 1 mL of 2 m MgCl_2_/20 mm Tris pH 7.4. The purified samples were desalted by Amicon Ultra‐0.5 centrifugal Filter devices (Millipore, Merck, Milan, Italy). A similar protocol was used to purify S and Z from plasma diluted 1 : 5 with 150 mm NaCl/20 mm Tris pH 7.4. Polymerization of purified proteins at 1–5 μm concentration in phosphate buffer pH 7.4 was induced either by heating at 55 °C for 4 h or by treating for 30 h at room temperature with 3 m GuHCl or 3 m urea.

### Sandwich ELISA

Ninety‐six‐well plates (Corning) were coated with antigen‐purified rabbit polyclonal anti‐AAT antibody (2 μg·mL ^−1^), washed (0.9% w/v NaCl, 0.05% v/v Tween20) and blocked with blocking buffer (PBS, 0.25% w/v bovine serum albumin, 0.05% v/v Tween20). Standards (purified M AAT or polymerized Z AAT) and samples were diluted in blocking buffer and incubated for 2 h at 37 °C. After washing, wells were incubated with either sheep anti‐AAT‐HRP (Abcam, Cambridge, UK) for AAT quantification, or with mAbs 2C1, 4B12 or 9C5 followed by anti‐mouse IgG horseradish peroxidase‐labelled antibody for affinity comparison, as indicated in the figure legends. HRP activity was measured by reaction with TMB and absorbance at 450 nm read on standard plate readers (Promega Glomax plate reader or Perkin Elmer Ensight).

### Recombinant expression and epitope mapping

Single‐cysteine variants of AAT with the C232S mutation were generated and expressed in *Escherichia coli* as described previously 20. Polymerization was induced at a concentration of 0.25 mg·mL ^−1^ in PBS with 0.5 mm Tween‐20 by heating at 55 °C for 1 h. Monomeric or polymeric protein was incubated at a concentration of 0.2 mg·mL ^−1^ and 35 μL volume with 0.5 : 1 molar ratio of monoclonal antibody (or in its absence) for 20 min at room temperature. CPM dissolved at a concentration of 100 mm in DMSO was rapidly diluted 1 : 5000 into PBS, and an equal volume was added to each sample. The conjugation reaction was monitored for 30 min using a SpectraMax M5 plate reader (Molecular Devices; em = 490 nm, ex = 370 nm). A Python/NumPy script was written to determine model‐free scaling factors along the intensity and time axes that optimally superimposed the curve in the presence of the antibody against the curve in its absence using least‐squares criteria.

### Molecular dynamics and modelling

Molecular dynamics simulations of the wild‐type and Trento AAT were performed with gromacs 5.0.4 49 based on the wild‐type AAT structure (PDB accession 1QLP); hydrogens were added, the system solvated with TIP3P water (≥ 10 Å between protein and box boundaries), and neutralized with Na^+^/Cl^−^ at a concentration of 150 mm. Simulations were conducted at 310 K using the amber99‐sb force field, the PME method for Coulomb interactions and a Lennard‐Jones potential (cut‐off of 10 Å) for short‐range interactions. After energy minimization, equilibration at constant volume for 100 ps and constant pressure for 100 ps, the production run was performed in the NPT ensemble (temperature control by velocity rescaling with a characteristic time of 0.1 ps; pressure control using the Parrinello–Rahman method with a time constant of 1 ps and a compressibility of 4.5∙10^−5^ bar^−1^). Hydrogen bonds were determined with a ≤ 3.5 Å acceptor–donor distance and ≤ 30 ° hydrogen‐donor‐acceptor angle. For each acceptor–donor pair, the number of hydrogen bonds was calculated every 2 ps in 10 independent 10‐ns simulations, and the average of these values was taken.

## Author contributions

EM, IF, JI, AF performed experiments, analysed data and wrote the article; RB, ML, MC, IH, SO, FG performed experiments and analysed data; EM, IF, DL, JI and AF planned the research; and all authors proof‐read and approved the manuscript.

## Conflict of interest

The authors declare that they have no conflict of interest with the work described herein.
